# A Self-Referenced Optical Intensity Sensor Network Using POFBGs for Biomedical Applications

**DOI:** 10.3390/s141224029

**Published:** 2014-12-12

**Authors:** Alberto Tapetado Moraleda, David Sánchez Montero, David J. Webb, Carmen Vázquez García

**Affiliations:** 1 Universidad Carlos III de Madrid, Displays and Photonics Applications Group, Electronics Technology Department, Avda Universidad 30, Leganés 28911, Spain; E-Mails: dsmontero@ing.uc3m.es (D.S.M.); cvazquez@ing.uc3m.es (C.V.G.); 2 Aston Institute of Photonic Technologies, Aston University, B4 7ET Birmingham, UK; E-Mail: d.j.webb@aston.ac.uk

**Keywords:** self-referenced fiber-optic sensor, WDM network, polymer optical fiber Bragg grating (POFBG), biomedical applications

## Abstract

This work bridges the gap between the remote interrogation of multiple optical sensors and the advantages of using inherently biocompatible low-cost polymer optical fiber (POF)-based photonic sensing. A novel hybrid sensor network combining both silica fiber Bragg gratings (FBG) and polymer FBGs (POFBG) is analyzed. The topology is compatible with WDM networks so multiple remote sensors can be addressed providing high scalability. A central monitoring unit with virtual data processing is implemented, which could be remotely located up to units of km away. The feasibility of the proposed solution for potential medical environments and biomedical applications is shown.

## 1. Introduction

Minimally invasive tools and sensors have become essential for medical diagnosis and surgery with the desire of not only serving at the same time to sense physiological parameters, but also being able to overcome biocompatibility concerns. Nowadays, there are different medical areas where fiber-optic sensors hold enormous potential such as in clinical biomechanics, particularly if *in-vivo* applications are pursued [1,2], as here there is a need to develop sensors for minimally invasive surgery procedures. Other important features include small size, light weight, geometrical flexibility, chemical inertness, electric and thermal insulation, and immunity to electromagnetic interference. Measurement principles mainly include the use of optical interferometers in multiple configurations (Sagnac, Michelson, Mach-Zehnder or Fabry-Perot), intensity-based fiber-optic sensor (FOS) and fiber Bragg gratings (FBGs). The first approach usually leads to extremely high sensitivity sensing solutions, but at the cost of very complex signal processing schemes, whereas the latter can also require a costly post-processing solution. In contrast, potentially low-cost intensity-based optical sensors modulate the power loss in response to changes in the desired measurand. These type of sensors have been successfully developed for use in MRI environments [3,4]. However, the main drawback of the intensity-based approach is the need for a self-referencing scheme in order to avoid undesirable perturbations in the optical power loss (due, for example, to changes in the source power) that can distort the measurements. Intensity-based FOS have been described for monitoring the intravascular blood pressure [5] and intracranial pressure (ICP) [6,7]. In these solutions, the self-reference property was achieved by a dual-beam technique, using a secondary optical fiber path, and then computing the ratio of both signals. Interesting applications of intensity-based FOS based on a bent optical fiber have also been developed to measure respiratory chest circumference changes [8] and the limb circumference change during occlusion plethysmography [9], respectively. Both sensors utilize the leakage of light from a fiber under mechanical perturbation. However, no self-referencing technique was applied, leading to noisy measurements which limited the range and resolution.

On the other hand, although polymer optical fibers (POFs) are seen as a lower cost alternative solution to silica-based short-distance optical links, they are also very attractive for exploitation in *in-vivo* sensing applications because they are inherently more biocompatible compared to their silica counterparts [10]. Moreover, the use of silica fiber is sometimes inappropriate due to the risks from breakages. No matter the case, the most common problem with these fiber-optic sensing solutions is the need of a fiber link between the point of detection which is in the vicinity of the patient and the read out unit which gives the required information. Areas most distant, considered as “remote”, from acute hospitals do not exceed 50 km and distances more than 10 km from a general practice service are extremely rare situations [11]. When continuous monitoring of critical parameters in day-to-day activities is required wireless portable interrogators can be cumbersome for both medical practitioners and patients [12] and a smart central monitoring unit for remote interrogation seems to be good choice.

On the other hand, the application of silica FBGs in medicine has already been described in detail in different reviews [13,14], and their introduction in clinical practice is just beginning [15]. There are also initial studies on biomedical applications focused on fiber-optic intensity-based sensors [16,17], the latter work focused on tapered fiber-optic biosensors, being capable nowadays to monitor a huge amount of measurands and still under development. In [18] a POF-based intensity fiber-optic sensor is reported, which is designed to monitor the human spine motions providing sensitivities around 1.3 dB/mm (optical power loss *vs.* fiber gap) and 0.24 dB/° (optical power loss *vs.* fiber tilt angle). An angular fiber-optic sensor with possible application in detecting the human extension's articulation is investigated in [19] providing sensitivities of 3 mV/° per angle of curvature. The detection of toluene dispersed in water was reported in [20] by swelling the POF cladding with a high density polyethylene (HDPE) to enhance the output light intensity change. It turned out a sensitivity of 2.3 dB/wt% with respect to the toluene concentration was obtainable.

Nowadays, the possibility of inscribing FBGs in POF is developed by using a special type of POF, the microstructured POF (mPOF) [21]. Those POF Bragg gratings try to take advantage of polymer benefits such as larger elastic limit, higher maximum strain limit and larger temperature and humidity responses compared to silica, while maintaining the benefits of FBG-based sensors. Nevertheless, limited effort has been directed towards synergizing biocompatible POF-based photonic sensing with the WDM interrogation method that allows multiplexing FBGs, with just a few exceptions [22]. The main underlying reason behind this lack of development is the mismatch between the optimum operating wavelength regions of POFs and the optical devices exploited for telecommunications purposes. The latter are developed for a wavelength region (C- and L-bands) totally unsuitable for POF-based transmission over medium-distances (hundreds of meters or greater) due to the high attenuation of poly(methyl methacrylate) (PMMA)-based POF of around 1 dB/cm at 1550 nm. Such high losses limit the practical length that POF can be used at this wavelength to typically less than tens of cm, requiring a connection stage at some stage to silica fiber to exploit the full capabilities of the WDM approach. Moreover, pigtailed sources, detectors, circulators and mux/demux devices are available off-the-shelf on silica fiber related technology. Consequently the fact that only a short length of POF can be used in the C- and L-bands requires a silica connecting lead to be used unless the sensor is mounted right next to the measurement unit, thus resulting in a very restricted sensing solution design criteria. This silica-POF combination has already been tested in [23] for a single sensor, where a short POFBG sensor section were glued to silica fibers on a POFBG accelerometer. Another approach is the use of POFBG devices near the optimum operating wavelength of the POF if longer fiber leads are going to be used, which can be more convenient for certain practical biomedical applications although preventing the use of relatively cheap optical devices designed and manufactured for silica fibers.

In this work, the feasibility of a hybrid silica-POF WDM network topology for addressing multiple self-referenced fiber-optic sensors is analyzed. The intention is to bridge the gap between the remote interrogation of multiple optical sensors and the advantages of using biocompatible POF-based sensors with low manufacturing cost, including those based on POFBGs. Another important attribute that will be discussed in this paper is the power budget analysis of the proposed topology as the POFBG needs to be integrated at some point with silica fiber related technology to make it useful.

## 2. Theory

**Figure 1:**
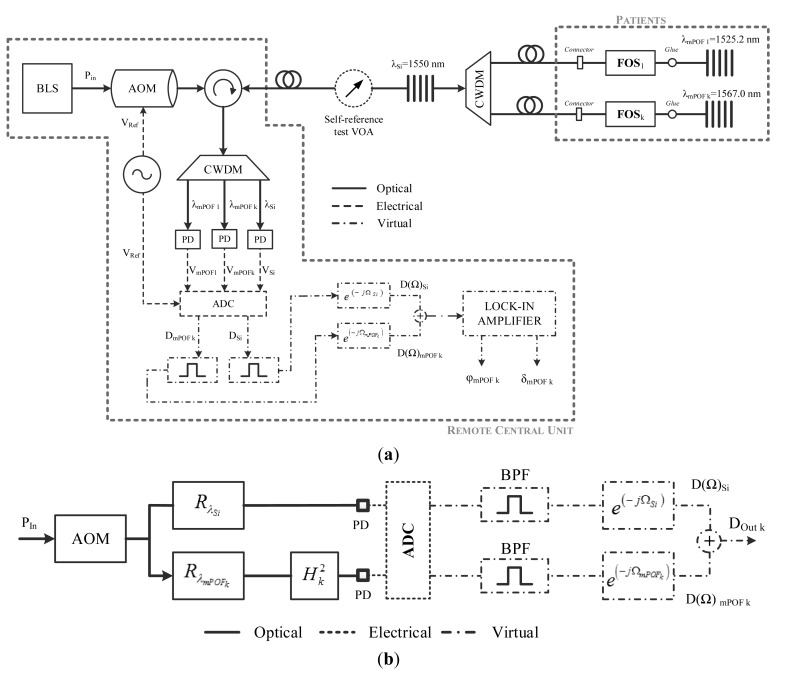
(**a**) Point-to-point self-referenced topology for generic remote sensing points; (**b**) Filter model of the configuration for a single remote sensing point including DAQ, bandpass filters (BPF) and virtual phase-shifts.

The proposed topology, depicted in Figure 1a, follows the frequency-based self-referencing technique for remotely addresing fiber-optic intensity sensors, as it is one of the most popular strategies for self-referencing schemes in the last decade. The use of resonant structures as basis of a self-referencing intensity type sensor has been widely identified in literature in an approach that is known as amplitude-phase conversion technique. In it, the optical power injected into the system is sine-wave-modulated. In the sensing head, a fraction of that power is not affected by the measurand, constituting a reference signal. The other fraction is intensity-modulated by the measured and constitutes the sensing signal. When both fractions are combined at the reception stage, it gives a resulting optical-power intensity sine wave. The phase of this signal, relative to the phase of the electrical signal that modulates the optical power emitted by the optical source, depends only on the optical loss induced in the sensor head by the measurand, including a constant factor determined by the length of the lead/return fiber. The evaluation of the phase allows information to be obtained about the measurand status; independently of the optical power fluctuations that can occur outside the sensor head thus performing a self-referenced measurement. The improvement of this configuration in comparison with other solutions [24–26] is the combination of silica and polymer FBGs for addressing multiple sensors at the remote points. In order to get a biocompatible system for medical applications, the usage of a single reference silica FBG and an improved remote reconfigurable virtual lock-in amplifier able to detect low signal variations provide extra features allowing the sensor interrogation.

A broadband light source (BLS) is externally modulated at a single frequency (f) by an acousto-optic modulator (AOM). The modulated broadband signal is launched into the remote sensing points via a broadband circulator and a Coarse Wavelength-Division Multiplexer (CWDM). Each remote sensing point consists of a sensing few-moded mPOF Bragg grating (mPOFBG) placed after the FOS. The proposed topology is compatible with any kind of POF-based intensity sensor as a FOS_i_ (see Figure 1) whereas the mPOFBGs employed provide the reflected back sensing channel to the central monitoring unit (which can be remotely located far away from the patient if necessary). The mPOFBGs are supposed to be located at the patient's vicinity, or even inside him/her if an invasive or *in-vivo* biomedical sensing application is considered. There is a silica-polymer glued connection in the FOS vicinity. Single silica FBG is located before the CWDM for reference purposes. The central wavelengths of the reference and sensing FBGs are λ_Si_ and λ_mPOF_, respectively. The broadband optical circulator receives the reflected multiplexing signals from the reference and the sensor channels, in which the sensor information is encoded. At the remote monitoring unit, the optical signal is demultiplexed by a CWDM device and distributed to an array of photodetectors (PD) by means of a data acquisition board (DAQ) which is used to convert the electrical signals from the photodetectors to digital signals together with a band-pass filter (BPF), used to eliminate noise from all signals at frequencies outside the system frequency. A phase-shift is applied to the reference and sensor digital signals. Finally, a virtual lock-in amplifier is used to interrogate all available sensor channels.

The block diagram for a single remote sensing point is shown in Figure 1b. The transfer function, using phasor representation, can be expressed as follows:

(1) $$\frac{{\text{P}}_{\text{out k}}}{{\text{P}}_{\ln}}=\alpha \cdot {\text{e}}^{\left(-\text{j}\cdot \Omega_{\text{Si}}\right)}\cdot \left[1+\beta_{\text{k}}\cdot {\text{e}}^{\left(-\text{j}\cdot (\Omega_{{\text{mPOF}}_{k}}-\Omega_{\text{Si}})\right)}\right]$$

with

(2) $$\alpha ={\text{m}}_{\text{Si}}\cdot {\text{d}}_{\lambda_{\text{Si}}}\cdot {\text{R}}_{\lambda_{\text{Si}}}$$

(3) $$\beta_{\text{k}}=\frac{{\text{m}}_{{\text{mPOF}}_{k}}\cdot {\text{d}}_{\lambda_{{\text{mPOF}}_{k}}}\cdot {\text{R}}_{\lambda_{{\text{mPOF}}_{k}}}\cdot {\text{L}}_{\text{CWD M}}\cdot {\text{L}}_{\text{mPOFBG}-\text{Si}}}{{\text{m}}_{\text{Si}}\cdot {\text{d}}_{\lambda_{\text{Si}}}\cdot {\text{R}}_{\lambda_{\text{Si}}}}\cdot H_{k}^{2}$$

where Ω_Si_and Ω_mPOF k_ are the phase shifts for the reference and each sensor signal. Parameters m_Si_, R_λ Si_ and d_λ Si_ are the modulation index, the reflectivity of the silica FBG and the photodetector responsivity, respectively, at the reference wavelength, whereas m_mPOF_, R_λPOF k_, and d_λ mPOF k_ are similar but for each sensor wavelength. H_k_ is the sensor power loss modulation and appears two times due to the reflective operation of the sensing structure. L_CWDM_ is the insertion loss for the CWDM. Finally, L_mPOFBG−Si_ are the mPOFBG insertion losses related to the reflectivity of the gratings, attenuation of the microstructured Polymer Optical Fiber (mPOF), multimode-singlemode silica fiber connection and silica-polymer glued connection.

The expression of the system in the Z-Transform domain can be identified with a digital Finite Impulse Response (FIR) filter as follows:

(4) $$\frac{{\text{P}}_{\text{Out k}}}{{\text{P}}_{\ln}}=\alpha^{\prime }\cdot \left(1+\beta_{\text{k}}\cdot {\text{z}}^{-1}\right)$$

with

(5) $$\alpha^{\prime }={\text{m}}_{\text{Si}}\cdot {\text{d}}_{\lambda_{\text{Si}}}\cdot {\text{R}}_{\lambda_{\text{Si}}}\cdot {\text{e}}^{\left(-\text{j}\cdot \Omega_{\text{Si}}\right)}$$

(6) $${\text{z}}^{-1}={\text{e}}^{\left(-\text{j}\cdot \Omega \right)}$$

**Figure 2:**
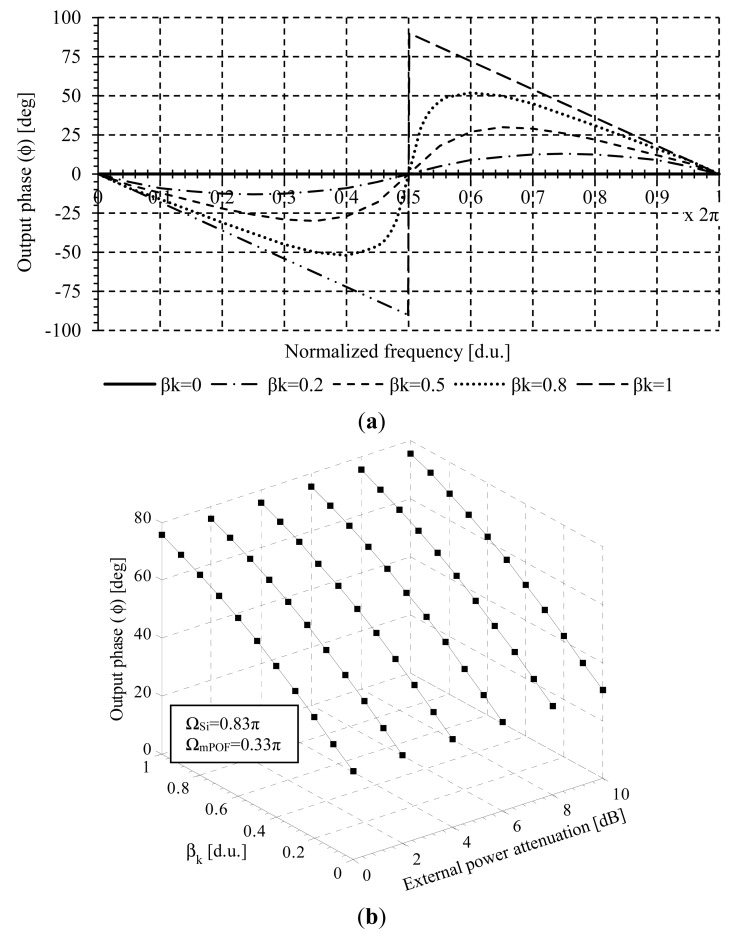
(**a**) Normalized phase response of the transfer function of the self-referencing configuration versus angular frequency for different values of β_k_; (**b**) Theoretical curves of the output phase ϕ_k_ versus β_k_ for different external power fluctuations at the reception stage.

The transfer function in the Z-Transform domain permits an easy study of the system frequency response in terms of generic design parameters [24]. In this approach, the phase shift difference Ω = Ω_mPOF_ − Ω_Si_ between the time domain reference and sensor signals represents, at the same time, the angular frequency of the digital filter. The normalized phase response *versus* the angular frequency (Ω) of the digital filter model is derived from Equation (4) and shown in Figure 2a for different values of β_k_. It is shown how the amplitude modulation is converted to phase variations. An antisymmetrical phase shape can be seen with regards to Ω = π. If β_k_ < 1, the phase response increases from zero to positive values as β_k_ takes greater values, being Ω > π, and the maximum values occur at angular frequencies tending to Ω = π^+^. For angular frequencies lower than Ω = π the phase response decreases from zero to negative values as β_k_ tends to one, and the peak value takes place around angular frequencies tending to π, being Ω = π^−^ the frequency of the zero.

Two measurement parameters can be defined for each remote sensing point, as reported in [25], but we will focus only on the output phase of the signal for different phase-shifts at the reception stage. This measurement parameter is derived from Equation (4) and is given by:

(7) $$\phi_{\text{k}}=\text{arctg}\left[\frac{-\left(\sin\Omega_{\text{Si}}+\beta_{\text{k}}\cdot \sin\Omega_{{\text{mPOF}}_{k}}\right)}{\left(\cos\Omega_{\text{Si}}+\beta_{\text{k}}\cdot \cos\Omega_{{\text{mPOF}}_{k}}\right)}\right]$$

The parameter Φ_k_ is insensitive to power fluctuations except for the sensor modulation (H_k_), thus aproviding a self-referenced measurement approach. A specific example of Φ_k_ versus the sensor losses β_k_ for different external power attenuations is shown in Figure 2b. Ω_Si_ = 0.83π and Ω_mPOF_ = 0.33π are the reference and sensor phase-shifts signals, respectively.

For a phase-shift fixed pair of values, the theoretical parameter (ϕ_k_) of the remote sensing point depends only on β_k_, which is insensitive to external power fluctuations that might take place in the optical link between the sensing point and the transmission stage. Moreover, the self-referencing parameter can be determined for any pair of phase-shift values providing flexibility to the measurement technique at the remote sensing network for any desired operation point.

## 3. Virtual Processing System

**Figure 3:**
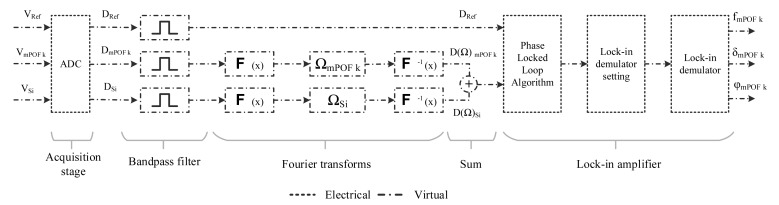
Block diagram of the computer software used to acquire and process the electrical signals.

In previous works [24–26] an analog delay by means an electronic circuit was used to get phase-shift at the reception stage. In this work, computer software based on a visual programming language has been designed to acquire and process the signals for the two sensing points. The software provides to the user a friendly environment and the ability to easily change the control parameters remotely. The block diagram of the computer software is shown in Figure 3. Four stages have been defined in the code: acquisition, filtering, phase shifter and lock-in amplifier.

The first stage uses a data acquisition assistant to convert the optical signals from the photodetectors and the lock-in reference signal to digital signals. The acquisition rate and the number of samples per channel was set to 16 kS/s and 8000 samples, respectively. The maximum and minimum signal input range was set to ±1 V in order to reduce the noise injected by the acquisition card.

The second stage uses a digital bandpass filter to eliminate noise from all acquired signals at frequencies outside the system frequency (f = 1 kHz). The designed filter uses a Butterworth topology with an order of 5. The low and high cutoff frequencies are fixed at 950 and 1050 Hz, respectively. These parameters can be changed using the graphical interface provided by the software.

After conditioning the signals, a phase shifter based on Fourier transforms is used to apply an independent delay to the digital reference and sensing signals. Then, both signals are added and introduced into the lock-in amplifier along with the digital lock-in reference signal.

Finally, a lock-in amplifier based on a graphical code [27,28] was used to obtain frequency, phase and amplitude of the added signal. The lock-in amplifier consists of three functions. The first function is a phase locked loop algorithm whose function is to measure the frequency and phase of the lock-in reference signal. The second function is used to internally calculate settings for the mixer and the low-pass filter in the demodulator function. Then a lock-in demodulator function extracts the frequency component from the added signal that uses the lock-in reference signal to specify the frequency and phase. In order to calculate the self-referencing parameter Φ_k_ one lock-in amplifier is necessary.

## 4. Measurements

**Figure 4:**
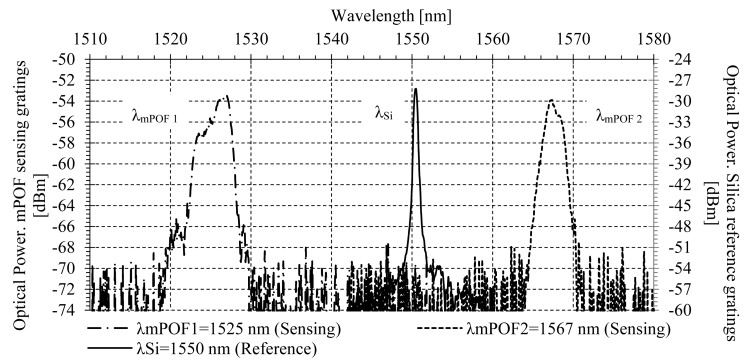
Optical spectrum in reflective operation of the mPOFBGs (sensing) and silica FBG (reference).

The network configuration shown in Figure 1a has been implemented using single mode silica fiber in order to experimentally validate the phase self-referencing parameter for two remote sensing points. A BLS modulated at f = 1 kHz by an acousto-optic modulator was employed to launch optical power into the configuration via a broadband circulator. One silica FBG was used for reference purpose, being placed after the broadband circulator and before the CWDM mux/demux. Its central wavelength and reflectivity were λ_Si_ = 1550 nm and 49%, respectively. A few-moded mPOFBG was used for each remote sensing point [21,29,30] located at the patient's vicinity. Their central wavelengths were λ_mPOF 1_ = 1525.2 nm for FOS_1_ and λ_mPOF 2_ = 1567.0 nm for FOS_2_, and their reflectivities were 27% and 36%, respectively. The attenuation of the mPOF was 0.82 dB/cm. The mPOF core and cladding diameter were 50 and 120 μm, respectively. It was made of three rings of holes on PMMA material. The optical spectrum of the 2-sensor network implemented is shown in Figure 4.

**Figure 5:**
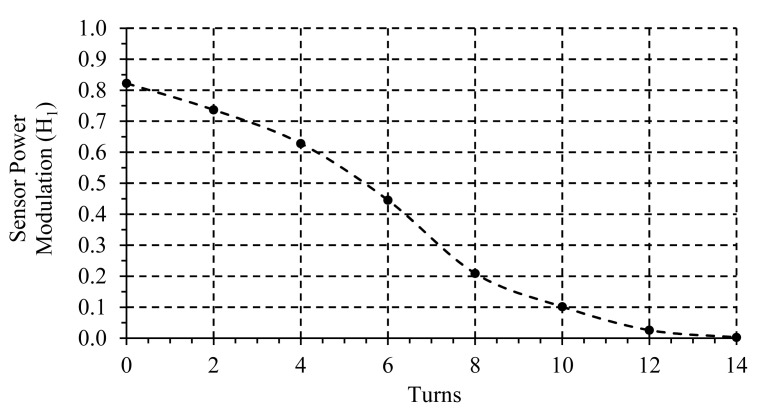
Calibration curve of the sensor loss modulation H_1_ emulated by means of a variable optical attenuator.

A single-mode Variable Optical Attenuator (VOA) was used to emulate the sensor response (FOS) and for calibration purposes. One example of the sensor loss modulation used to calibrate the configuration is shown in Figure 5.

The reflected signals were demultiplexed by a CWDM and detected by three amplified InGaAs detectors. The amplifier gain was fixed at 70 dB for all measurements. A 14-bit low-cost DAQ was used to convert the electrical signals from the photodetectors to digital signals. Computer software was used to implement the bandpass filter, the phase-shifts and the lock-in amplifiers at the reception stage. One virtual lock-in amplifier per sensor was used to obtain the self-referencing parameter Φ_k_, with i = 1,2.

### 4.1. Self-Reference Measurements

**Figure 6:**
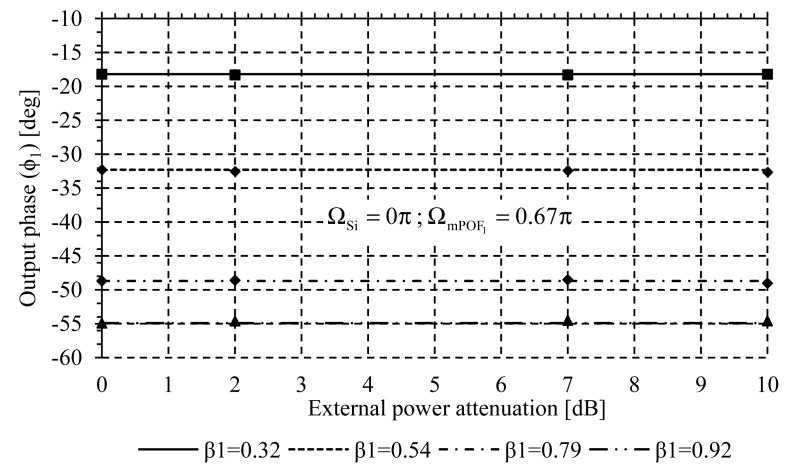
Output phase φ_1_ self-reference test *versus* noise power fluctuations for different values of sensor losses at the remote sensing point addressed by λ_mPOF 1_.

The self-reference property was tested by inducing power fluctuations in the modulated optical source through a VOA. From Figure 6, it is demonstrated that the self-reference system was able to regulate at 0.46% output phase after inducing 10 dB of power fluctuations. A normalization procedure has been used to span β_k_ from Equation (3) to a range with a maximum value of 1, and output phase is scaled proportionally. This procedure applies to all reported measurements.

### 4.2. Crosstalk Analysis

**Figure 7:**
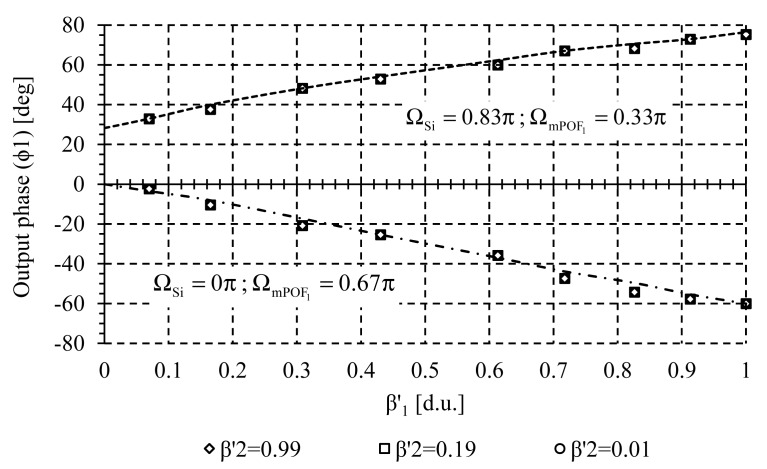
Crosstalk related measurements. Output phase parameter *versus* sensor loss at channel λ_mPOF 1_ (FOS_1_) for different values of sensor loss at channel λ_mPOF 2_ (FOS_2_).

**Figure 8:**
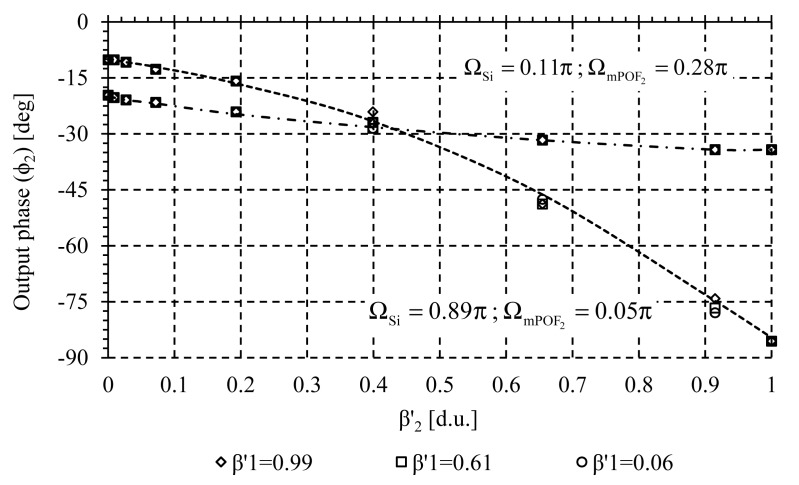
Crosstalk related measurements. Output phase parameter *versus* sensor loss at channel λ_mPOF 2_ (FOS_2_) for different values of sensor loss at channel λ_mPOF 1_ (FOS_1_).

Crosstalk analysis was carried out to measure the possible interference between adjacent channels during the measuring process. Several measurements of the self-referencing parameter Φ_1_(FOS_1_) at wavelength λ_mPOF 1_ were taken for different values of the sensor loss modulation β_2_(FOS_2_) at wavelength λ_mPOF 2_. Both virtual delays, Ω_Si_ and Ω_mPOF k_ are selected to achieve positive and negative incremental system response, with high linearity and sensitivity. Experimental result is shown in Figure 7. Similar results were obtained when monitoring Φ_2_ (FOS_2_) at wavelength λ_mPOF 2_ when sensor loss β_1_ was changed, see Figure 8. In both cases no crosstalk was noticed, so both sensors can be interrogated simultaneously without mutual interference because of the high channel isolation of the CWDM demultiplexer.

## 5. Discussion

In this article, a hybrid silica-POF WDM network topology for addressing multiple self-referenced fiber-optic POF-based sensors has been proposed. As expected, its performance in terms of crosstalk between sensors and its self-referencing property have been validated. However, system factors such as the sensitivity, the resolution and the power budget which limits the remote interrogation distance reachable or the maximum sensor insertion losses, must be further investigated.

From Equation (3), if the modulation index and the photodetector responsivity are considered to be similar at reference and sensor wavelength the new sensor sensitivity is given by:

(8) $$\frac{\partial \beta_{\text{k}}}{\partial \text{A}}=2\cdot \text{H}\cdot \frac{{\text{R}}_{\lambda_{\text{mPOFk}}}}{{\text{R}}_{\lambda_{\text{Si}}}}\cdot {\text{L}}_{\text{CWDM}}\cdot {\text{L}}_{\text{mPOFBG}-\text{Si}}\cdot \frac{\partial \text{H}}{\partial \text{A}}$$

where A is the magnitude to be measured and ∂H/∂A was implemented in the experiments using a VOA. From Equation (8), we see that the different losses are the dominant factor and they should be kept as low as possible.

On the one hand, the most limiting components in the system's resolution are the transimpedance amplifier gain for the detectors. The InGaAs detectors used in this experiment offer a noise value of 1.5 mV_RMS_ at a gain of 70 dB. With this noise value, the system resolution, considering the detected output power, is 1.7 × 10^−2^ dB. Using another amplifier detector with a lower NEP, the system resolution would be limited by the data acquisition card to a value of 6.5 × 10^−3^ dB. These resolution values are far below those provided by most of the POF intensity-based sensing solutions reported in literature, and particularly for biomedical applications.

**Figure 9:**
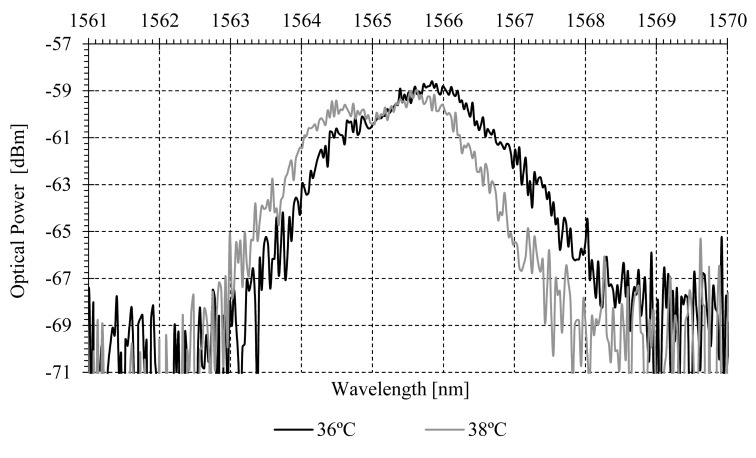
Optical spectrum in reflective operation for the mPOFBGs *versus* temperature.

By additionally monitoring each central mPOFBG wavelength shift within the range of each CWDM channel, temperature can be measured, apart from the parameter under test. Temperature characterization of one sensing point can be seen in Figure 9.

One of the disadvantages of using PMMA-based FBGs is their aptitude for water absorption, and PMMA FBG sensors have a significant cross-sensitivity to humidity. These problems might be reduced by using FBGs based on TOPAS fiber [31]. The humidity sensitivity of a TOPAS-based FBG is ∼64 times smaller than for an equivalent FBG manufactured in PMMA fiber at 1565 nm [32]. On the other hand, TOPAS fiber has the same high attenuation as its PMMA counterpart in the 1550 nm spectral region and also needs to be glued at the end of a singlemode silica fiber lead for being connected to other optical devices.

Another important issue with POFBGs is their cross-sensitivity to temperature. The self-referenced technique uses a POFBG placed at the vicinity of the patients, which is affected by the patient's temperature. The displacement of the Bragg wavelength with temperature is following analyzed in order to cope the wavelength spacing between the CWDM mux/demux channels. The two channels used in the measurements have a central wavelength of 1571.3 for FOS_1_ and 1531.1 for FOS_2_, with passband widths 15.9 and 16.4 nm, respectively. The mPOF Bragg wavelength variation with temperature is −60 pm/°C. The Full Width at Half Maximum (FWHM) of the gratings are 5.8 for FOS_1_ and 4.8 nm for FOS_2_. The minimum and maximum temperature that a human body can stand without dying are ∼20 and ∼41.1 °C [33], respectively. Considering the sensitivity and the human body temperature range, the wavelength shift (Δλ_mPOFShift_) is ∼1 nm well within the CWDM mux/demux pass band width. In a general design, to overcome the cross-sensitivity of the sensing gratings, the central wavelength of the mPOFBGs used should fulfil the following condition:

(9) $$\lambda_{{\text{mPOF}}_{i}}+\Delta \lambda_{\text{mPOF Shift}}+\frac{\text{FWH}{\text{M}}_{{\text{mPOF}}_{i}}}{2}<\lambda_{{\text{CWDM}}_{i}}+\frac{\text{B}{\text{W}}_{{\text{CWDM}}_{i}}}{2}$$

where λ_CWDMi_ and BW_CWDMi_ are the central wavelength and the pass-band width of each mux/demux channel, respectively. FWHM_mPOFi_ is the FWHM parameter of the grating used in each channel. The above estimation of the wavelength shift can be considered to have a negligible impact on the parameters previously defined in Equation (3) for a real-case scenario thus allowing simple sensor interrogation.

Table 1 shows the optical power budget analysis of the remote sensing topology. P_m_ is the optical power launched into the system by the broadband light source (BLS), L_Devices_ computes the total power loss of light travelling in both senses of direction (due to the reflective topology) including the acousto-optic modulator, optical circulator, silica FBG, mux/demux CDWMs, adaptors and FOS. The maximum power variation of the FOS is fixed at 6 dB, high enough to cover any biomedical input magnitude span. P_out_ is the photodiode noise-equivalent power (NEP) figure of merit and the value provided in Table 1 refers to the minimum measurable optical power, in terms of both NEP and bandwidth. L_CWDM_ is the CWDM insertion loss. L_mPOFBG−Si_ are the mPOFBG insertion losses related to the reflectivity of the gratings (R_λmPOF k_), attenuation of the mPOF fiber (α_mPOF_ = 0.82 dB/cm [34], multimode-singlemode silica fiber connection (L_(SM−MM)Si_ = 1.56 dB) and silica-polymer glue union (L_Si−mPOF_ ≈ 5 dB) [30].

Computing the power budget at the most restrictive sensing wavelength in terms of distance reachable, a maximum length of 11 km could be obtained. However, the latter can be easily improved and extended to the access network domain (up to 20 km) by launching more optical power into the system, using optical devices with better insertion loss performance or using a more efficient technique to connect mPOFBGs. In comparison to other configurations that use splitters, a power budget improvement in more than 15 dB for a 16-sensor network can be achieved [23].

Considering the available optical power, the proposed method could provide a remote monitoring service unit fully compliant for short-reach networks (typically less than 1 km), *i.e.*, Local Area Networks (LANs) and in-building/in-hospital networks. Indeed it is suitable for medium reach-distances (typically up to 10 km) with application in inter-hospital networks or to provide a convergent all-optical and straightforward connection between patient's homes and a general practice service for telemedicine purposes. However, the latter can also be easily provided by including a wireless transmission at any point of the optical fiber link beyond the remote monitoring unit. Nevertheless, the above distances are unbeatable if an all-POF-based optical network is intended to be deployed and a hybrid approach should be considered.

**Figure 10:**
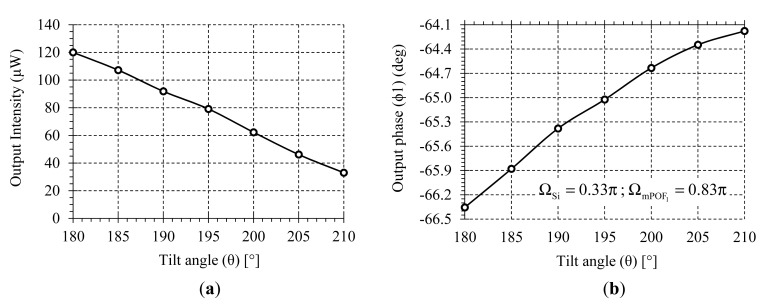
(**a**) Light intensity *versus* fiber tilt angle [18]; (**b**) Output phase parameter *versus* fiber tilt angle at channel λ_mPOF 1_ (FOS_1_).

Finally, the proposed self-referenced sensor network has been evaluated with an intensity-based optical sensor for the measurement of bend angles in the spines of human physiotherapy patients. The received light intensity decreases as the gap between emitting and receiving fibers and/or the bending angle between them increase. The output power measurements versus the applied tilt angle is extracted from [18] and shown in Figure 10a. Using Equations (3) and (7), the output phase parameter versus tilt angle at channel λ_mPOF 1_ (FOS_1_) has been calculated for a specific set of virtual delays, as shown in Figure 10b.

As demonstrated before, the propose topology for self-reference multiple intensity fiber optic sensors is able to regulate at 0.46% output phase after inducing 10dB of power fluctuations. Further development of a spine bending sensor [35] introduces a single sensor self-referenced technique by adding more fibers and allowing 0.55% output signal fluctuations after inducing only ∼1.4 dB. Other important feature of the proposed technique in this work is the possibility to address multiple self-referenced intensity optical fiber sensors providing great flexibility and easy reconfiguration.

## 6. Conclusions

The need for an optical fiber link between the remote sensing area at the vicinity of the patient and the measuring unit may be the most important concern when employing inherently biocompatible POF intensity-based optical sensors. The feasibility of a hybrid silica-POF WDM network topology for addressing multiple self-referenced fiber-optic POF-based sensors is demonstrated. The intention is to bridge the gap between the remote interrogation of multiple optical sensors and the advantages of using biocompatible POF, including those based on mPOFBGs. One self-reference parameter has been tested to be robust to 10 dB power fluctuations. Proper selection of both virtual delays can lead to linear responses at high sensitivities.

The proposed topology has high scalability and power budget enhancement in comparison with all POF based solutions as it uses off-the shelf WDM devices with low insertion losses and a lock-in detection scheme. It also provides great flexibility and easy reconfigurability due to the use of virtual instrumentation solutions. By additionally monitoring each central POFBG wavelength shift within the range of each CWDM channel, temperature changes can be tracked.

The self-referenced solution uses a low-cost 14-bit DAQ board which offers a resolution of1.7 × 10^−2^ dB. The resolution and the number of sensors to be interrogated are limited by the number of analog channels and the resolution provided by the DAQ board, but easily improved. The proposed topology provides a central remote monitoring unit that can be located several km away from the patient's location, where the sensors are placed. It is shown the potential of the proposed technique to be useful in self-referencing multiple intensity optical sensors for measuring bend angles in the spines of human physiotherapy patients.

## Figures and Tables

**Table 1.** Optical power budget analysis of the proposed hybrid silica-POF WDM remote sensing scheme.

**mPOFBG Central Wavelength**	**BLS Output Power, dBm** P_in_	**Devices Insertion Losses, dB** L_Devices_	**CWDM Insertion Loss, dB** L_CWDM_	**mPOFBG Insertion Losses, dB** L_mPOFBG−Si_**(Note 1)**	**Photodetector Sensitivity, dBm** P_Out_ **(Note 2)**
λ_1_ = 1525 nm	−17.3	30.2	1.8	15.1	−66.6
λ_2_ = 1567 nm	−15.8	30.2	1.8	14.6	−66.6

Notes: 1. Including R_λ mPOF k_; 2. Considering the amplified gain of 70 dB, the noise-equivalent power (NEP) and the bandwidth of the amplified InGaAs detector of $2\times 10^{-12}\text{W}/\sqrt{\text{Hz}}$ and 12 kHz, respectively.

## References

[1] Arkwright J., Blenman N., Underhill I., Maunder S., Szczesniak M., Dinning P., Cook I. (2009). In-vivo demonstration of a high resolution optical fiber manometry catheter for diagnosis of gastrointestinal motility disorders. Opt. Express.

[2] Webb D.J., Jones S., Zhang L., Bennion I., Hathaway M., Jackson D. (2000). First in-vivo trials of a fiber Bragg grating based temperature profiling system. J. Biomed. Opt..

[3] Polygerios P., Puangmali P., Schaeffer T., Razavi R., Seneviratne L.D., Althoefer K. Novel miniature MRI-compatible fiber-optic force sensor for cardiac characterization procedures.

[4] Taffoni F., Formica D., Saccomandi P., Pino G.D., Schena E. (2013). Optical fiber-based MR-compatible sensors for medical applications: An overview. Sensors.

[5] Lekholm A., Lindström L. (1969). Optoelectronic transducer for intravascular measurements of pressure variations. Med. Biol. Eng..

[6] Hollingsworth-Fridlund P., Vos H., Daily E. (1988). Use of fiber-optic pressure transducer for intracranial pressure measurements: A preliminary report. Heart Lung J. Crit. Care.

[7] Wald A., Post K., Ransohoff J., Hass W., Epstein F. (1977). A new technique for monitoring epidural intracranial pressure. Med. Instrum..

[8] Babchenko A., Khanokh B., Shomer Y., Nitzan M. (1999). Fiber optic sensor for the measurement of respiratory chest circumference changes. J. Biomed. Opt..

[9] Stenow E.N., Oberg P. (1993). Venous occlusion plethysmography using a fiber-optic sensor. IEEE Trans. Biomed. Eng..

[10] Webb D.J. Polymer photonic crystal fibre sensor applications.

[11] Jordan H., Roderick P., Martin D., Barnett S. (2004). Distance, rurality and the need for care: Access to health services in South West England. Int. J. Health Geogr..

[12] Witt J., Narbonneau F., Schukar M., Krebber K., De Jonckheere J., Jeanne M., Kinet D., Paquet B., Depre A., D'Angelo L.T. (2012). Medical textiles with embedded fiber optic sensors for monitoring of respiratory movement. IEEE Sens. J..

[13] Mishra V., Singh N., Tiwari U., Kapur P. (2011). Fiber grating sensors in medicine: Current and emerging applications. Sens. Actuators A Phys..

[14] Kalinowski H.J. Fiber Bragg grating applications in biomechanics.

[15] Ho S.C.M., Razavi M., Nazeri A., Song G. (2012). FBG sensor for contact level monitoring and prediction of perforation in cardiac ablation. Sensors.

[16] Roriz P., Ramos A., Santos J.L., Simões J.A. (2012). Fiber optic intensity-modulated sensors: A review in biomechanics. Photon. Sens..

[17] Leung A., Shankar P.M., Mutharasan R. (2007). A review of fiber-optic biosensors. Sens. Actuators B Chem..

[18] Zawawi M., O'Keeffe S., Lewis E. (2013). Plastic optical fibre sensor for spine bending monitoring. Sensors.

[19] Cherbi L., Mehenni M., Aksas R. (2004). Conception and realization of an angular optical sensor. Microw. Opt. Technol. Lett..

[20] Fujii Y., Honma S., Morisawa M., Muto S. Development of new optical fiber toluene sensor.

[21] Barton G., van Eijkelenborg M.A., Henry G., Large M.C., Zagari J. (2004). Fabrication of microstructured polymer optical fibres. Opt. Fiber Technol..

[22] Berghmans F., Geernaert T., Sulejmani S., Thienpont H., Steenberge G.V., Hoe B.V., Dubruel P., Urbanczyk W., Mergo P., Webb D.J. Photonic crystal fiber Bragg grating based sensors—opportunities for applications in healthcare.

[23] Stefani A., Andresen S., Yuan W., Herholdt-Rasmussen N., Bang O. (2012). High sensitivity polymer optical fiber-Bragg-grating-based accelerometer. IEEE Photon. Technol. Lett..

[24] Montalvo J., Araújo F., Ferreira L., Vázquez C., Baptista J.M. (2008). Electrical FIR filter with optical coefficients for self-referencing WDM intensity sensors. IEEE Photon. Technol. Lett..

[25] Montalvo J., Frazão O., Santos J.L., Vázquez C., Baptista J.M. (2009). Radio-frequency self-referencing technique with enhanced sensitivity for coarse WDM fiber optic intensity sensors. J. Lightw. Technol..

[26] Montero D., Vázquez C., Baptista J., Santos J., Montalvo J. (2010). Coarse WDM networking of self-referenced fiber-optic intensity sensors with reconfigurable characteristics. Opt. Express.

[27] Corporation N.I. How to Measure Small Signals Buried in Noise Using LabVIEW and Lock-in Amplifier Techniques. http://www.ni.com/white-paper/5613/en/.

[28] Corporation N.I. Multi Channel Count Lock-in Amplifier with Simulated Data or NI-4472 and DAQmx. http://www.ni.com/example/29532/en/.

[29] Dobb H., Webb D.J., Kalli K., Argyros A., Large M.C., van Eijkelenborg M.A. (2005). Continuous wave ultraviolet light-induced fiber Bragg gratings in few-and single-mode microstructured polymer optical fibers. Opt. Lett..

[30] Zhang C. (2011). Fibre Bragg Grating in Polymer Optical Fibre for Applications in Sensing. Ph.D. Thesis.

[31] Johnson I.P., Yuan W., Stefani A., Nielsen K., Rasmussen H.K., Khan L., Webb D.J., Kalli K., Bang O. (2011). Optical fibre Bragg grating recorded in TOPAS cyclic olefin copolymer. Electron. Lett..

[32] Yuan W., Khan L., Webb D.J., Kalli K., Rasmussen H.K., Stefani A., Bang O. (2011). Humidity insensitive TOPAS polymer fiber Bragg grating sensor. Opt. Express.

[33] Trautner B.W., Caviness A.C., Gerlacher G.R., Demmler G., Macias C.G. (2006). Prospective evaluation of the risk of serious bacterial infection in children who present to the emergency department with hyperpyrexia (temperature of 106 F or higher). Pediatrics.

[34] Carroll K.E., Zhang C., Webb D.J., Kalli K., Argyros A., Large M.C. (2007). Thermal response of Bragg gratings in PMMA microstructured optical fibers. Opt. Express.

[35] Zawawi M.A., O'Keeffe S., Lewis E. (2013). Plastic Optical Fibre Sensor for Spine Bending Monitoring with Power Fluctuation Compensation. Sensors.

